# Influence of Olive Extracts on the Expression of Genes Involved in Lipid Metabolism in Medaka Fish

**DOI:** 10.3390/molecules24173068

**Published:** 2019-08-23

**Authors:** Luis Torró-Montell, Ernesto Cortés-Castell, Elia Sirvent-Segura, Carmen Veciana-Galindo, Vicente Gil-Guillén, Mercedes Rizo-Baeza

**Affiliations:** 1 Biopartner S.L., Alcoy, 03800 Alicante, Spain; 2 Department of Pharmacology, Pediatrics and Organic Chemistry, Miguel Hernández University, 03550 San Juan de Alicante, Spain; 3 Department of Clinical Medicine, Miguel Hernández University, 03550 San Juan de Alicante, Spain; 4 Department of Nursing, University of Alicante, 03690 San Vicente del Raspeig, Spain

**Keywords:** olive, polyphenol extracts, lipogenic genes, lipolytic genes, medaka fish

## Abstract

**Aims**. To assess the possible effect of polyphenol-rich olive extracts on lipid metabolism in medaka fish by quantifying the expression of lipogenic and lipolytic genes. **Materials and methods**. Adult medaka fish were maintained in tanks for five days with five extracts at 0.01% in water, causing obesity through a diet rich in carbohydrates, with a control group maintained in water with a normal diet. The extracts contained polyphenols ranging between 7 and 116 mg/g (oleuropein, hydroxytyrosol) with an antioxidant power of 2–13 mmol of 2,4,6-tri(2-pyridyl)-1,3,5-triazine/100 g. After five days, the fish were sacrificed and the hepatic mRNA and its complementary DNA were extracted by reverse transcription. Complementary DNAs were quantified for three lipolytic and three lipogenic genes by real-time PCR. The relative gene expression was calculated from the amplification curves in reference to the control group. **Results**. The expression of genes involved in lipolysis, including *peroxisome proliferator-activated receptor-±, acyl-CoA oxidase 1*, and *carnitine palmitoyltransferase 1*, were clearly decreased in fish subjected to an obesogenic diet, and this situation could not be reversed in fish maintained with polyphenol-rich extracts. In contrast, *lipogenic fatty acid synthase, acetyl-CoA carboxylase 1*, and *sterol regulatory element-binding protein 1* genes increased considerably with the obesogenic diet and reverted to the normal state with the olive extracts. The effect was not dependent on the total polyphenol content, the specific oleuropein or hydroxytyrosol concentration, or the antioxidant power, suggesting a synergistic effect. **Conclusion**. Olive polyphenols, acting as anti-lipogenic agents, have a positive effect on lipid metabolism, but their mechanism in each gene is different according to the extract, which supports synergistic mechanisms with the different proportions of polyphenols and accompanying phytochemicals in each extract.

## 1. Introduction

Lipid metabolism is closely related to obesity and overweight. Accordingly, an increase in fat deposits is produced either by the inactivation of lipolysis, favoring the accumulation of fat or reducing its elimination, or by the activation of lipogenesis, caused by, among other factors, an increase in energy intake, producing the same results. These processes are closely related to indicators of some metabolic syndrome factors. Certain polyphenols of plant origin have been shown to confer healthful properties beyond the simple nutrient content of the foods that contain them, with these foods being designated as functional foods. Among these are foods derived from olive trees. For example, the PREDIMET study (Prevention with Mediterranean Diet Study) concluded that a diet enriched with olive oil significantly reduces cardiovascular risk [1]. Similarly, in the ESCARVAL study (Valencian Cardiometabolic Study), the most important protective factor against cardiovascular disease was found to be higher HDL-Cholesterol [2], as seen in populations consuming a healthy Mediterranean diet including olive oil. 

Studies have proven the effect of polyphenols in reducing fat deposits in rats by reducing adiponectin and the PPARγ pathway [3]. Other studies have pointed to the anti-inflammatory power of polyphenols as a mechanism for preventing or reducing obesity [4], to their complex action as regulators of hunger-satiety signals [5], and to an increase in the capacity of polyphenols to defend mitochondria from oxidative stress and thus contribute to cellular energy homeostasis [6]. With polyphenol-rich olive extracts, our research group has previously shown in mouse fibroblasts a decrease in fat accumulation, in the number of adipocytes, and in the expression of leptin and PPARγ genes [7]. 

Medaka fish are an animal model with a greater than 70% genomic homology with humans and have therefore been used in food and drug study trials [8,9,10]. Specifically, the medaka fish shares the mechanisms of digestion, absorption, transport, and oxidation of lipids with higher mammals [11]. 

The objective of this study was to evaluate the possible effect of polyphenol-rich olive extracts on lipid metabolism using the medaka fish as a model. Accordingly, we analyzed the expression of genes involved in lipolysis, including *peroxisome proliferator-activated receptor-⍺* (*PPAR*⍺), *acyl-CoA oxidase 1* (*ACO1*), and *carnitine palmytoyl transferase 1* (*CPT1*), as well as genes involved in lipogenesis such as *fatty acid synthase* (*FAS*), *acetyl-CoA carboxylase 1* (*ACC1*), and *sterol regulatory element-binding protein 1* (*SREBP1*) [12]. 

## 2. Results

### Expression of Genes Involved in Lipolysis

Table 1 presents the relative gene expression values of the lipolytic genes, which show a clear decrease in expression in overfed fish compared to control fish.

The results of the analysis of the expression of genes involved in lipogenesis are presented in Table 2.

It should be noted that understanding the effects of polyphenol-rich olive extracts on metabolism must be considered in terms of the previous impact of overfeeding on the expression of these genes.

PPAR⍺ belongs to a family of nuclear receptor proteins that act as regulating factors in the expression of various genes involved in functions, including lipid metabolism, that bind to specific DNA sites when binding to free fatty acids [13,14]. In lipid metabolism, PPAR⍺ activation increases the expression of genes encoding enzymes that promote fatty acid oxidation. In our study, fish in which obesity was induced by an enriched diet showed a clear decrease to 0.28 compared to 1.10 in normally fed control fish (*p* = 0.006). This decrease was not significantly reversed by any of the extracts; in fact, it was increased by two of the extracts (E2 and E4). Therefore, PPAR⍺ does not appear to be the possible pathway for polyphenol-rich olive extracts (Table 1).

ACO1 is the enzyme involved in the first step of fatty acid oxidation through β-oxidation. An obesogenic environment reduced gene expression by nearly half (*p* = 0.045), a situation that was significantly reversed by E1 (*p* = 0.035). Although E3 and E4 also reversed gene expression, these reversals were not significant due to the large standard deviation (Table 1).

The CPT1 enzyme binds long-chain fatty acids to carnitine to be transported into the mitochondria, where they are oxidized. In an obesogenic environment, this enzyme’s gene expression was reduced by approximately half (1.37±1.38 to control *vs* 0.66±0.66; *p* = 0.011 for the obesogenic control). This reduction was significantly reverted to the gene expression levels of the normally fed controls by E2 (1.24±0.58; *p* = 0.035) and E5 (0.97 ± 0.71; *p* = 0.047). Although also appeared to increase gene expression, these increments did not reach significance due to the large dispersion in the standard deviations. Thus, the extract E1 doesn’t restore the expression of the gene with respect to the obesogenic control (0.70 ± 0.43; *p* = 0.468), while the extracts E3 and E4 increase although not significantly said expression (0.91 ± 0.57; *p* = 0.104 and 0.93 ± 0.86; *p* = 0.461, respectively), (Table 1).

FAS is the enzyme complex responsible for acid synthesis, with palmitic acid as its final product. Its gene expression was highly increased, by about 90 times (*p* < 0.001), in controls subjected to an enriched diet. This situation was reversed with all the extracts, although without reaching complete normality (control values). The descending order was E3 > E5 > E2 > E4 > E1, which is not in line with the total polyphenols, antioxidants, or oleuropein and hydroxytyrosol present in these extracts (Table 2).

The enzyme ACC1 catalyzes the irreversible conversion of acetyl-CoA to malonyl-CoA, and plays an essential role in the regulation of fatty acid synthesis and degradation in response to the energy status of the body. In fish subjected to an obesogenic diet, gene expression increased about nine times (*p* < 0.001). This situation reverted to normal with E2 and E3 (*p* < 0.001) and decreased, but without reaching normal levels, with E1 (*p* = 0.003) (Table 2).

Finally, SREBP1 is a transcription factor required for proper lipid homeostasis, regulating the transcription of the LDL-cholesterol receptor gene and the synthesis of glycogen and neoglycogenic genes [15]. In obese fish, gene expression decreased by about 25% and was nearly significant (*p* = 0.083). This situation was reversed, and the expression was even increased with E1 (*p* = 0.025), with no differences observed with the other extracts (Table 2).

## 3. Discussion

### 3.1. Summary

The induced obesity decreased the expression of all the lipolytic genes compared to the control fish without overfeeding. With the extracts, the expression varied greatly, though always between the values of the obese and normally fed fish, with the effect on each gene varying for each extract. In all three lipogenic genes, a higher expression was observed among the obese than among the normally fed fish. The values among the overfed fish returned to a normal expression in the presence of all the extracts, but with different degrees of efficiency and with no correlation with the effect of each extract on the lipolytic genes, nor with their polyphenol content or antioxidant power. This shows a variability in the mechanisms of action of the extracts and, furthermore, that this general anti-lipolytic function does not depend on the content of a single polyphenol and antioxidant power, but rather on a synergistic effect. 

### 3.2. Strengths and Limitations 

The main strength of this study is the research idea, in which the possible effect of polyphenol extracts on lipid metabolism was tested, using the expression of three mRNAs involved in lipogenic and lipolytic processes, thus embracing a wide range of possible mechanisms of action.

Possible limitations include the small number of fish used and the wide range of dispersion of the results, which in some cases led to clear increases or decreases in gene activation, but without significant differences. In some cases, therefore, the effect was not fully confirmed. Finally, it should be noted that the effect analyzed cannot refer to a single component of the extracts, since a complex mixture was used without purification of its polyphenols and possible bioactive phytochemical components. However, this possible limitation and the absence of a link between the effects analyzed and the majority of the main polyphenols or the antioxidant power point to synergistic effects already described by other authors.

### 3.3. Comparison with the Literature

The action of citrus flavonoid glycosides has been described, such as that of scutellarin which activates the PPARγ receptor, showing a strong hypolipidemic, antioxidant, and liver-protective activity that could be attributed to their regulatory activity in the PPARγ/PGC-1α-Nrf2 signaling pathway [16]. Another citrus flavonoid, nobiletin, shows effects on lipogenesis, inhibiting the accumulation of liver lipids by acting on lipogenic factors such as SREBP-1c and FAS [17]. Hesperidin also modulates the inflammatory response and antioxidant status through the downregulation of PPARγ and Bcl-2 expression in mice [18]. Curcumin, quercetin, and atorvastatin also regulate the expression of lipogenic genes, such as CPT1, and generally protect against liver fibrosis by reducing the accumulation of liver fat [19]. Quercetin also enhances cholesterol absorption by hepatocytes, with beneficial effects on cholesterol homeostasis and atherogenesis [20].

Similarly, hydroxytyrosol, present in extra virgin olive oil, increases the activity of PPARα and may reduce liver alterations induced in mice [21]. Hydroxytyrosol can also correct metabolic deficiencies in diet-induced obesity in mice by correcting the aberrant expression of genes involved in hepatic lipogenesis (SREBP1, ACC, FAS, and Stearoyl-CoA desaturase-1), decreasing hepatic steatosis from induced obesity [22]. A similar effect was demonstrated by the same authors with 6-gingerol, a phenolic component of ginger [23].

Apigetrine, a flavonoid found in many plant leaves and seeds, has antimutagenic, anticancer, antioxidant, and anti-inflammatory properties, but also reduces levels of lipogenic gene expression and inhibits the adipogenesis of 3T3-L1 preadipocytes in the early stage of adipogenesis [24]. 

In our study, as described above, the effect of olive extract polyphenols on the decrease in lipogenic gene expression previously induced by overfeeding was more evident. The data confirm our earlier studies in which we observed a decrease in the formation of fatty bodies and a decrease in the expression of PPARγ and leptin genes in the differentiation of adipocytes in the presence of a polyphenolic extract from olive pits in mouse fibroblasts [7]. In addition, it has been seen that the action of hydroxytyrosol must be modulated by other phytochemicals present in the mixture, since its activity once purified is lower than that of the mixture of extracts [25]. This is consistent with the lack of correlation of the effects found by our group with the concentration of the individual main polyphenols, total polyphenols, or the antioxidant power of the extracts we used. 

### 3.4. Applicability and Future Lines of Research

The results obtained demonstrate the beneficial action of olive products on lipid metabolism and present the possibility of using olive extracts as nutritional supplements, conferring on these products the qualification of functional foods. It is important to determine which substances or mixtures of these substances have the greatest anti-lipogenic potential. Plans for a series of future lines of research include extending the research to mammals and, finally, to humans.

## 4. Material and Methods

### 4.1. Subjects

The medaka fish (*Orzyas latipes*) was used as a model, as previous studies have shown that the lipid metabolism in these fish and that in higher mammals are practically identical [11]. 

### 4.2. Procedure

Six adult male medaka fish were selected per tank, using one fish tank for each study group. The fish were maintained for five days with the different extracts (Table 3) at 0.01% in water, and obesity was induced through a carbohydrate-rich diet (Gemma micro 300 ZF; ingredients: fish meal, lecithin, wheat gluten, dried seaweed, fish oil, maize starch, vitamins, and minerals; crude fat: 14%). The details of the extracts are proprietary and cannot be disclosed.

The control group was comprised of fish maintained only in water and without an obesogenic diet. After five days, the six fish of each group were sacrificed. The liver was extracted and homogenized and the DNA was eliminated by DNAse I (Qiagen). The sacrifice was made through anesthesia by immersion in Tricaine 50 mg/l until fish stop responding to touch, followed by quick decapitation. Using the RNeasy extraction kit (Qiagen), mRNA was extracted and used as a template by transcriptase (High-Capacity cDNA reverse transcription kit, Applied Biosystems) to obtain complementary DNA (cDNA) from the mRNA present in the liver. The cDNA was amplified employing the specific primers for each of the previously mentioned genes using real-time (quantitative) PCR, the primers described in Table 4, and the PrimeScript RT (Perfect Real Time) real-time amplification kit (Takara Clontech). Amplification of β-actin cDNA was used as a reference for the entire process. All trials were performed in triplicate.

### 4.3. Validation of Primers

To evaluate the correct amplification and specificity of the primer pairs for each of the genes analyzed, the melting curves were obtained for each pair, showing efficient amplification, with a single peak (amplicon) at the melting point and an adjusted temperature range between 85 and 90 °C, indicating a high specificity of the primers and suitability for the application of qRT-PCR as they did not show primer dimer structures. Fluorescence also increased linearly during amplification to 20–30 cycles. 

### 4.4. Calculation of Gene Expression

From the amplification curves and using the control group as a reference, the relative value of the expression of the different genes analyzed was determined. We used the Pfaffl equation: R = (E_target_)^ΔCPtarget(control-sample)^/(E_reference_)^ΔCPreference(control-sample)^, where E is the efficiency of each gene, target refers to the gene of study, and reference refers to the housekeeping gene. The efficiencies obtained were: *PPAR*⍺ 100%, *ACO1* 92%, *CPT1* 93%, *FAS* 92%, *ACC1* 94%, *SREBP1* 92%, and *β-actin* 97%. Means and standard deviations were obtained and a comparison was made using the student’s *t*-test. 

### 4.5. Ethical Considerations

The research complied with the established legal and ethical procedures and was carried out under PG-NBP-13 included in the R + D + i Quality Management System (ref. 166002) of the Neuron Bio Company, ENAC certification. The procedure was approved by the Committee of Ethics of Animal Experimentation of the Research Foundation of the University Hospital and Polyclinic La Fe (Valencia, Spain) (Code number: ES462500001013).

## 5. Conclusions

Olive polyphenols appear to have beneficial effects on lipid metabolism, acting as anti-lipogenic agents, but the modification of the mechanism expression in each gene differs according to the extract used. Since there is no correlation between the observed effects and the concentration of the majority polyphenols and antioxidant power of the extracts, it seems that these effects are produced by synergistic mechanisms between the different proportions of polyphenols and the phytochemicals that accompany them in each extract.

## Figures and Tables

**Table 1:** Relative expression of lipolytic genes compared to the control.

Medium	*PPAR*⍺	*p*-Value	*ACO1*	*p*-Value	*CPT1*	*p*-Value
Control	1.10 ± 0.86	0.006	1.10 ± 0.80	0.045	1.37 ± 1.38	0.011
Control OB	0.28 ± 0.18	-	0.67 ± 0.45	-	0.66 ± 0.66	-
E1	0.20 ± 0.14	0.447	1.55 ± 1.18	0.035	0.70 ± 0.43	0.468
E2	0.09 ± 0.06	0.040	0.54 ± 0.68	0.174	1.24 ± 0.58	0.035
E3	0.11 ± 0.07	0.043	1.14 ± 1.01	0.292	0.91 ± 0.57	0.104
E4	0.52 ± 0.39	0.274	1.21 ± 1.10	0.254	0.93 ± 0.86	0.461
E5	0.32 ± 0.22	0.625	0.50 ± 0.20	0.290	0.97 ± 0.71	0.047

**Table 2:** Relative expression of lipogenic genes compared to the control.

Medium	*FAS*	*p*-Value	*ACC1*	*p*-Value	*SREBP1*	*p*-Value
Control	1.44 ± 1.40	<0.001	1.50 ± 1.46	<0.001	1.00 ± 0.03	0.083
Control OB	94.70 ± 63.60	-	9.40 ± 6.47	-	0.75 ± 0.51	-
E1	32.90 ± 18.97	0.008	2.68 ± 2.77	0.003	1.58 ± 1.10	0.025
E2	21.26 ± 22.87	0.001	1.07 ± 1.25	<0.001	0.36 ± 0.20	0.061
E3	7.26 ± 10.37	<0.001	1.18 ± 0.79	<0.001	0.52 ± 0.39	0.332
E4	27.93 ± 21.62	0.001	6.23 ± 5.96	0.207	0.61 ± 0.20	0.735
E5	19.95 ± 16.73	<0.001	6.02 ± 4.33	0.091	0.40 ± 0.20	0.104

**Table 3:** Characteristics of the olive aqueous extracts used.

Extract	Total Polyphenols (mg/g Extract)	Total Antioxidants (mmol TPTZ/100 g) *	Oleuropein (% *w/w*)	Hydroxytyrosol (% *w/w)*
E1	23	3	1.12	0.2
E2	68	8	0.5	0.03
E3	7	2	<0.003 (LD)	<0.001 (LD)
E4	116	13	1.24	10.17
E5	49	5	1.27	1.99

TPTZ, 2,4,6-*tri*(2-pyridyl)-1,3,5-triazine; LD, limit of detection. * FRAP assay.

**Table 4:** List of complementary DNA (cDNA) of the amplified genes and primers used.

Gene	Forward Primer (5′-3′)	Reverse Primer (3′-5′)
*PPAR* *⍺*	TCTTGAGTGTCGGGTGTGTG	CGGTAGAGCCCACCATCTT
*ACO1*	GCTCAGCTTTACAGCCTTGG	GGACGATTCCCTAACGATCA
*CPT1*	ATGTCTACCTCCGTGGACGA	CAAGTTTGGCCTCTCCTTTG
*FAS*	GACGCTTCAGGAAATGGGTA	GGACAGGAACCGGACTATCA
*ACC1*	GAGTGACGTCCTGCTTGACA	ACCTTTGGTCCACCTCACAG
*SREBP1*	CCCAACCAGATGAGGAGAAA	AGGACTTTTGTGCTGCTCGT
*β-actin*	CTGGACTTCGAGCAGGAGAT	CTGGAAGGTGGACAGAGAG

*ACC1*: *acetyl-CoA carboxylase 1*; *ACO1*: *acyl-CoA oxidase 1*; *CPT1*: *carnitine palmytoyl transferase 1*; *FAS*: *fatty acid synthase*; *PPAR*⍺: *peroxisome proliferator-activated receptor-⍺*; *SREBP1*: *sterol regulatory element-binding protein 1*.
